# A unified coarse-grained model of biological macromolecules based on mean-field multipole–multipole interactions

**DOI:** 10.1007/s00894-014-2306-5

**Published:** 2014-07-15

**Authors:** Adam Liwo, Maciej Baranowski, Cezary Czaplewski, Ewa Gołaś, Yi He, Dawid Jagieła, Paweł Krupa, Maciej Maciejczyk, Mariusz Makowski, Magdalena A. Mozolewska, Andrei Niadzvedtski, Stanisław Ołdziej, Harold A. Scheraga, Adam K. Sieradzan, Rafał Ślusarz, Tomasz Wirecki, Yanping Yin, Bartłomiej Zaborowski

**Affiliations:** 1 Faculty of Chemistry, University of Gdańsk, ul. Wita Stwosza 63, 80-308 Gdańsk, Poland; 2 Laboratory of Biopolymer Structure, Intercollegiate Faculty of Biotechnology, University of Gdansk and Medical University of Gdansk, ul. Kładki 24, 80-922 Gdańsk, Poland; 3 Baker Laboratory of Chemistry and Chemical Biology, Cornell University, Ithaca, NY 14853-1301 USA; 4 Department of Physics and Biophysics, Faculty of Food Sciences, University of Warmia and Mazury in Olsztyn, Michała Oczapowskiego 4, 10-719 Olsztyn, Poland

**Keywords:** Coarse-graining, Mean-field approach, Multipole–multipole interactions, Proteins, Nucleic acids, Polysaccharides

## Abstract

A unified coarse-grained model of three major classes of biological molecules—proteins, nucleic acids, and polysaccharides—has been developed. It is based on the observations that the repeated units of biopolymers (peptide groups, nucleic acid bases, sugar rings) are highly polar and their charge distributions can be represented crudely as point multipoles. The model is an extension of the united residue (UNRES) coarse-grained model of proteins developed previously in our laboratory. The respective force fields are defined as the potentials of mean force of biomacromolecules immersed in water, where all degrees of freedom not considered in the model have been averaged out. Reducing the representation to one center per polar interaction site leads to the representation of average site–site interactions as mean-field dipole–dipole interactions. Further expansion of the potentials of mean force of biopolymer chains into Kubo’s cluster-cumulant series leads to the appearance of mean-field dipole–dipole interactions, averaged in the context of local interactions within a biopolymer unit. These mean-field interactions account for the formation of regular structures encountered in biomacromolecules, e.g., α-helices and β-sheets in proteins, double helices in nucleic acids, and helicoidally packed structures in polysaccharides, which enables us to use a greatly reduced number of interacting sites without sacrificing the ability to reproduce the correct architecture. This reduction results in an extension of the simulation timescale by more than four orders of magnitude compared to the all-atom representation. Examples of the performance of the model are presented.

**Figure:**
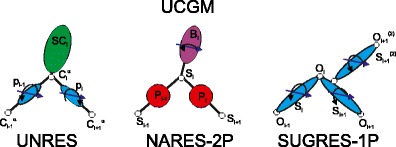
Components of the Unified Coarse Grained Model (UCGM) of biological macromolecules

Components of the Unified Coarse Grained Model (UCGM) of biological macromolecules

## Introduction

Coarse-graining is the method of choice when simulating large systems [1–3]. Particular research effort has been directed toward the development of coarse-grained models of biological macromolecules such as proteins [3–17], nucleic acids [3, 18–33], carbohydrates [34–36], and biological assemblies, such as lipid bilayers [37, 38]. In this approach, a number of atoms are merged into single interaction sites, and the solvent surrounding the system is usually treated at the mean-field level in the form of a continuous medium. The main purpose of such an approach is to enable us to run simulations at time and size scales that are orders of magnitude greater than possible using the all-atom approach [39]. This is a great advantage despite the exponential growth of computing power in recent years (especially that due to the introduction of graphical processor units [40], which capitalized on parallel computations at a scale unknown before, and the very recent construction of the ANTON supercomputer by Shaw and coworkers [41], which made ab initio simulations of the folding of small proteins at the detailed atomistic scale possible [42]). On the other hand, recent work suggests that coarse graining can also be used as a means to understand the rules behind the formation of macromolecular structure and macromolecular dynamics [43–45].

Constructing coarse-grained force fields is a much greater challenge than constructing all-atom force fields; the physical foundations of coarse-grained force fields were discovered only relatively recently [1, 46]. These force fields are divided into two main categories: knowledge-based and physics-based. Knowledge-based force fields are derived based on statistics determined from structural databases [4], while physics-based force fields relate all-atom energy surfaces to effective coarse-grained energy surfaces [13]. Physics-based force fields can, in turn, be divided into neoclassical force fields, in which the functional form is copied from that of all-atom force fields (e.g., the very widely applied MARTINI force field [38]), and those that are based on the understanding of a coarse-grained force field as a potential of mean force in which the degrees of freedom that are not omitted from the model have been integrated out [1, 46].

Based on our understanding of coarse-grained force fields as potentials of mean force, over the last 20 years we have been developing our physics-based united residue (UNRES) model of polypeptide chains [46–54]. To derive the force field in a systematic and consistent way, we developed a method of factorizing the PMF in contributions arising from smaller fragments of the system (thereby making it computable and transferable). These factors can also be expanded into the Kubo cluster-cumulant series [55], thereby enabling us to obtain analytical expressions for the respective terms, especially multibody terms, which are derived in other force fields in a heuristic manner [4]. Another very important feature of the UNRES model is that it emphasizes the role of electrostatic interactions involving polar peptide groups, which are represented as the mean-field interactions between peptide-group dipoles. These mean-field interactions are the main factors responsible for the formation of regular α-helical and β-sheet structure in proteins [44, 46].

The success of the UNRES model prompted us to extend the philosophy of constructing coarse-grained models to other biological macromolecules, namely nucleic acids and polysaccharides, and to produce the unified coarse-grained model (UCGM) for all these three classes of macromolecules that occur in all living organisms as building materials and perform a variety of functions. Very recently, using the very same concept, we extended the UNRES model to the nucleic acid united residue two-point model (NARES-2P), in which one interaction site per nucleotide is the phosphate group and the second is the nucleic acid base merged with its sugar ring. These sites serve as the polar units which interact via mean-field dipole–dipole interactions. Despite its simplicity, the NARES-2P model reproduces the double-helical structures of small DNA and RNA molecules and the melting thermodynamics of small DNA molecules surprisingly well [56]. We have also extended the treatment to polysaccharides, to produce the sugar united residue one-point model (SUGRES-1P).

In this paper, the theory behind the unified coarse-grained model is presented, and its components—the UNRES, NARES-2P, and the as-yet unpublished SUGRES-1P models—are described. Results of simulations performed using the three force fields are presented, and perspectives on their unification into one system—which will be able to treat not only the structures and dynamics of the isolated components but also interactions and composites of them, such as glycans—are outlined.

## Methods

### The unified coarse-grained model of biological macromolecules

**Fig. 1:**
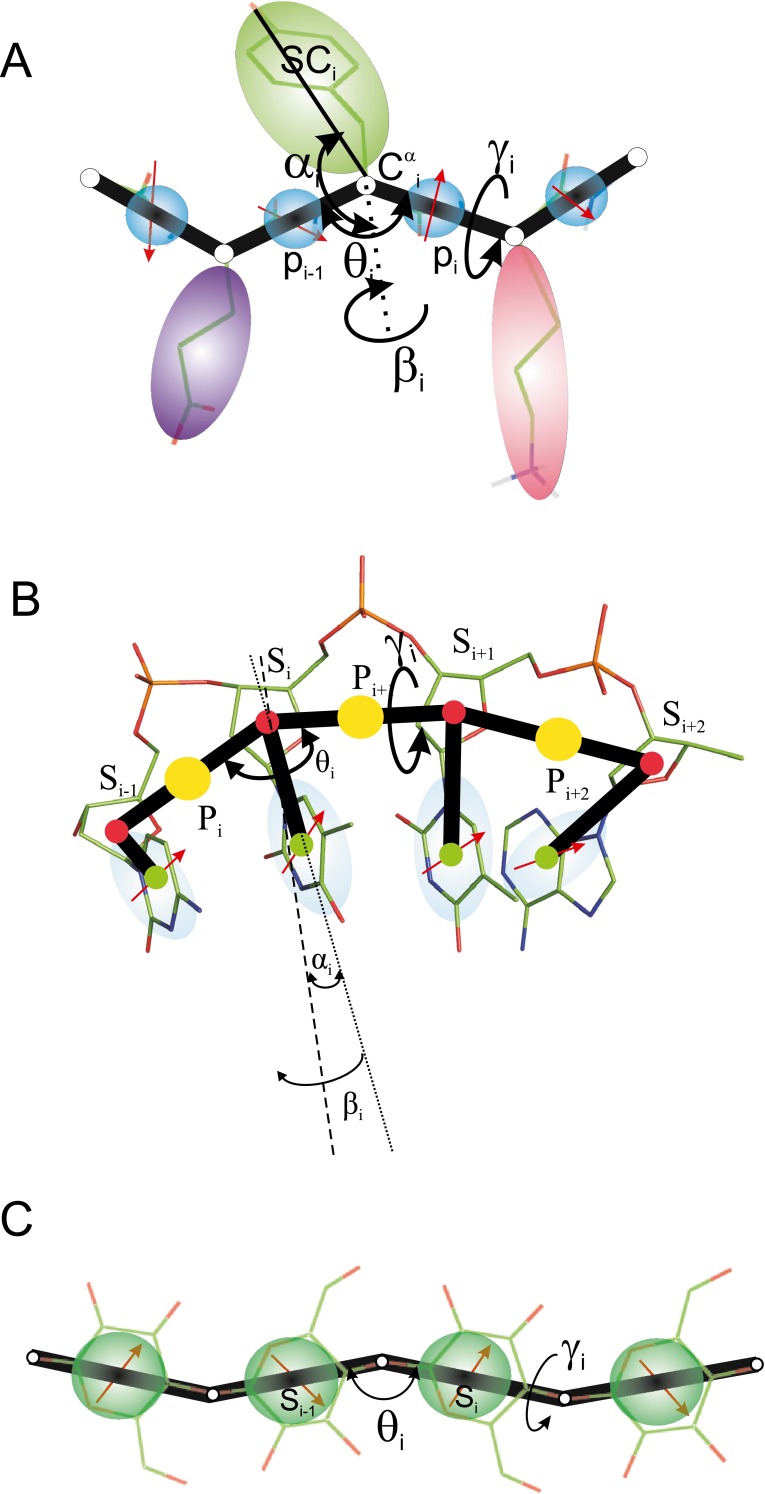
**a**–**c** The components of the unified coarse-grained model of biological macromolecules (UCGM). **a** UNRES (proteins), **b** NARES-2P (nucleic acids), **c** SUGRES-1P (polysaccharides). The polar interaction sites bearing point dipoles (depicted as *red arrows*) are colored *blue* and the virtual bonds are shown as *thick black lines*. It should be noted that the dipoles are not fixed but rotate about the virtual-bond axes to give average potentials. For nucleic acids (panel **b**), the dipole moments of the purine bases are approximately parallel to the S⋯B virtual-bond (rotation) axis; they are approximately perpendicular for pyrimidine bases, which explains the geometry of Watson–Crick pairing [56]. All-atom chains are superposed on coarse-grained representations for better illustration. In the UNRES model of polypeptide chains (**a**), the interaction sites are side chains, represented as ellipsoids of revolution of different sizes (*SC*) attached to the corresponding α-carbon atoms (represented by *small open circles*), and peptide-bond centers (*p*). The equilibrium length of a peptide bond is 3.8 Å for the *trans* and 2.8 Å for the *cis* configuration. For the *i*th residue, the geometry of the respective chain fragment can be described using virtual-bond angles *θ*_*i*_, virtual-bond dihedral angles *γ*_*i*_, and the polar angles *α*_*i*_ and *β*_*i*_. In the NARES-2P model of the nucleotide chain, the interaction sites are phosphate groups (*P*), represented by *yellow circles*, and the nucleic acid bases fused with sugar rings, represented by *ellipsoids*, with their geometric centers at the Bs (*green circles*). The Ps are located halfway between two consecutive sugar atoms. The backbone virtual-bond angles *θ* and the virtual-bond dihedral angles *γ*, as well as the polar angles *α* and *β* that define the orientation of the sugar-base vector with respect to the backbone, are also shown. *Small red circles* represent the sugar-ring centers (*S*) which serve as geometric points. In the SUGRES-1P model of the polysaccharide chains, the interaction sites are the sugar rings (*S*), represented by *blue transparent spheres*, while *white circles* represent the glycosidic oxygen atoms (*O*) that serve as anchor points

As mentioned in the “Introduction,” the unified coarse-grained model of biological macromolecules is a generalization of the approach taken when designing the UNRES model for proteins [46–54]. It assumes that (i) a biopolymer unit has an easily distinguishable polar site with a charge distribution represented by a point multipole and that (ii) the mean-field interactions between the polar sites, averaged in the context of local interactions, determine the symmetry of regular structures. The components of the model—the UNRES, NARES-2P, and SUGRES-1P models for proteins, nucleic acids, and polysaccharides, respectively—are visualized in Fig. 1.

In the following subsections, we will outline the method used to derive the coarse-grained force field through cluster-cumulant-function expansion of the potential of mean force of the system developed in our earlier work [46, 49, 51]. We will then provide short descriptions of the components of the model.

### Potential of mean force of a coarse-grained system and its expansion into Kubo cluster-cumulant functions

In our approach for polypeptide chains [46], we assume that the effective energy function of a system is the potential of mean force (PMF), also termed the restricted free energy function (RFE), with all degrees of freedom that are lost when passing from the all-atom to the coarse-grained model averaged out. These neglected degrees of freedom include solvent degrees of freedom, side-chain rotation angles, and the dihedral angles *λ* for rotation of the peptide groups about the C^α^⋯C^α^ virtual bonds. The solvent degrees of freedom are usually averaged out explicitly using Monte Carlo (MC) or molecular dynamics (MD) simulations, or implicitly using data from the PDB [57]. Thus, the variables describing the geometry of the macromolecule–water system are divided into two sets: the *primary* variables (**X**), which describe the coarse-grained degrees of freedom, and the less important or *secondary* variables (**Y**) that are averaged out. In general, the RFE [*F*(**X**)] is expressed as

1 $$ F\left(\mathbf{X}\right)=- RT \ln \left\{\frac{1}{V_{\mathbf{Y}}}{\displaystyle {\int}_{\varOmega_{\mathbf{Y}}} \exp \left[- E\left(\mathbf{X};\mathbf{Y}\right)/ RT\right]{\displaystyle {\mathrm{dV}}_{\mathbf{Y}}}}\right\}, $$

where ${V}_{\mathbf{Y}}={\displaystyle {\int}_{\varOmega_{\mathbf{Y}}}{\displaystyle {\mathrm{dV}}_{\mathbf{Y}}}}$, *E*(**X**; **Y**) is the original (all-atom) energy function, *R* is the universal gas constant, *T* is the absolute temperature, *Ω*_**Y**_ is the region of the **Y** subspace of variables over which the integration is carried out, and V _**Y**_ is the volume of this region.

**Fig. 2:**
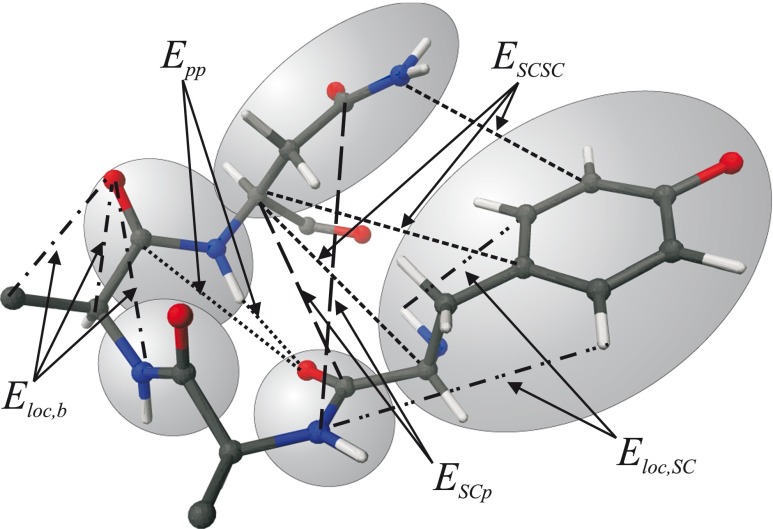
The splitting of the interaction energy into component energies, as illustrated using a fragment of a polypeptide chain. *SC* denotes a side chain and *p* denotes a peptide group. The atoms of two side chains and three peptide groups of the portion of the polypeptide chain shown in the picture are embedded in *shaded ellipsoids*. For the sake of clarity, only some of the interactions are shown and the water molecules are not included. The terms *E*_AB_ denote the interaction energies between the atoms of sites A and B (e.g., *E*_SCp_ denotes the interactions between a side chain and a peptide group), while *E*_loc,b_ and *E*_loc,SC_ denote the energies that contribute to local-interaction energies within the backbone and the side-chain part of a given residue, respectively. Reproduced with permission from Figure 2 of [48]

To identify the effective energy terms, the all-atom energy *E*(**X**; **Y**) is expressed as a sum of component energies, each of which is either the sum of energies within a given unit (the *local interaction energies*) or between given units (the *long-range interaction energies*), as given by Eq. 2 below. The RFE (Eq. 1) is decomposed into factors, each of which is a *Kubo cluster-cumulant function* [55], as expressed by Eq. 3 below and illustrated in Fig. 2.

**Fig. 3:**
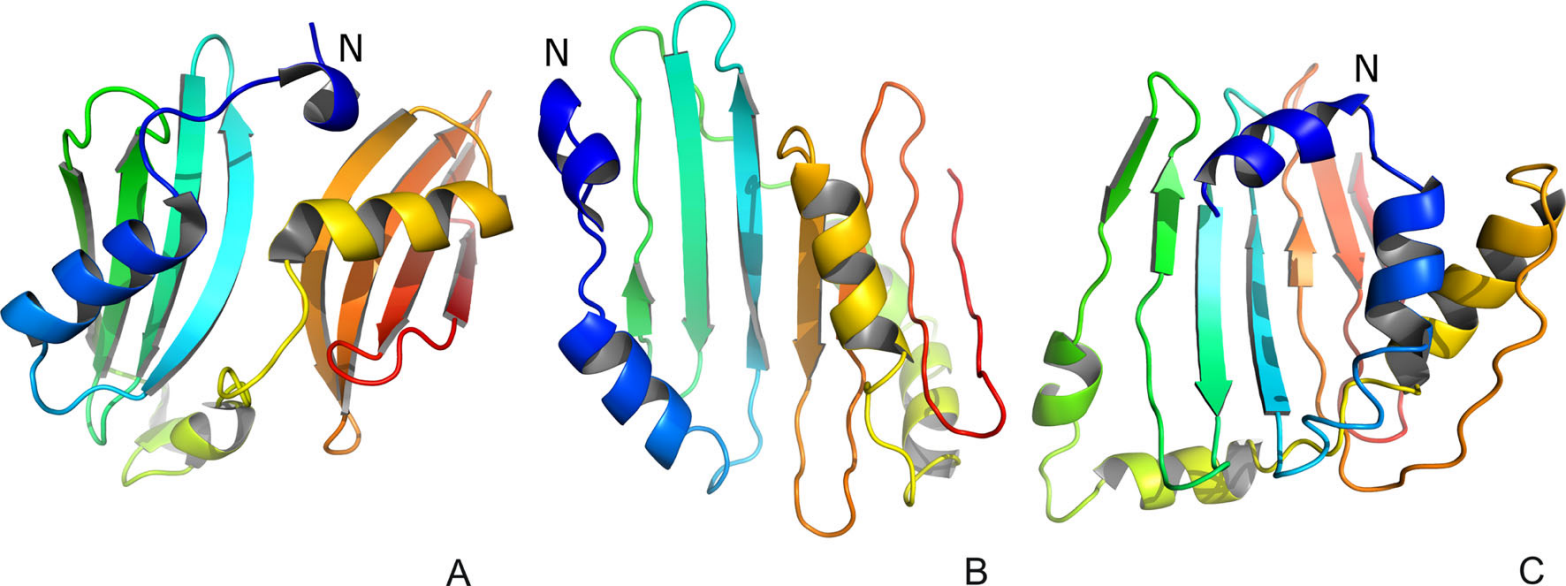
**a** The experimental 4EXR structure of CASP10 target T0663. **b** Our model 1. **c** Our model 4. The N-termini in panels **a–c** are labeled *N*. The values of GDT_TS are 23.19, 31.98, and 42.80 for model 1 of the whole protein and its domains D1 and D2, respectively, and 22.04, 31.98, and 40.15 for model 4 of the whole protein and its domains D1 and D2, respectively. The respective GDT_TS values of the models with the highest GDT_TS values submitted to CASP are 42.93 (model 1 from group 27), 68.61 (model 3 from group 27), and 98.20 (model 4 from group 27). The drawings of the structures were produced with PYMOL (http://www.pymol.org). Reproduced with permission from Figure 1A-C of [84]

2 $$ E\left(\mathbf{X};\mathbf{Y}\right)={\displaystyle \sum_{i=1}^n}{\upvarepsilon}_i\left(\mathbf{X};{\mathbf{z}}_{\mathbf{i}}\right), $$

where ε_*i*_(**X**; **z**_**i**_) is the *i*th component energy, **z**_**i**_ contains the secondary degrees of freedom on which ε_*i*_ depends, and *n* is the number of energy components.

3 $$ F\left(\mathbf{X}\right)={\displaystyle \sum_i{f}_i^{(1)}\left(\mathbf{X}\right)}+{\displaystyle \sum_{i< j}{f}_{i j}^{(2)}\left(\mathbf{X}\right)}+{\displaystyle \sum_{i< j< k}{f}_{i j k}^{(3)}\left(\mathbf{X}\right)}+\dots +{\displaystyle \sum_{i_1{<}_2\dots <{i}_n}{f}_{i_1{i}_2\dots {i}_n}^{(n)}\left(\mathbf{X}\right)} $$

The factors are expressed as

4 $$ \begin{array}{c}\hfill {f}_{i_1{i}_2\dots {i}_k}^{(k)}={\left\langle \left\langle {\upvarepsilon}_{i_1}{\upvarepsilon}_{i_2}\dots {\upvarepsilon}_{i_k}\right\rangle \right\rangle}_f={\displaystyle \sum_{l=1}^k{\displaystyle \sum_{\begin{array}{c}\hfill {i}_{m_1}<{i}_{m_2}<\dots <{i}_{m_l}\hfill \\ {}\hfill {m}_i\in \left[1.. k\right]\hfill \end{array}}{\left(-1\right)}^{k- l}{F}_{i_{m_1}{i}_{m_2}\dots {i}_{m_l}}^{(l)}}}=\hfill \\ {}\hfill ={\displaystyle \sum_{l=1}^k{\displaystyle \sum_{\begin{array}{c}\hfill {i}_{m_1}<{i}_{m_2}<\dots <{i}_{m_l}\hfill \\ {}\hfill {m}_i\in \left[1.. k\right]\hfill \end{array}}{\left(-1\right)}^{k- l}\left\langle \left\langle {\upvarepsilon}_{i_{m_1}}{\upvarepsilon}_{i_{m_2}}\dots {\upvarepsilon}_{i_{m_l}}\right\rangle \right\rangle }}\hfill \end{array} $$

where

5 $$ {F}_{i_1,{i}_2,\dots {i}_k}^{(k)}\left(\mathbf{X}\right)\equiv \left\langle \left\langle {\upvarepsilon}_{i_1}{\upvarepsilon}_{i_2}\dots {\upvarepsilon}_{i_k}\right\rangle \right\rangle =-\frac{1}{\beta} \ln \left\{\frac{1}{V_{{\mathbf{y}}_I}}{\displaystyle \underset{\varOmega_I}{\int } \exp \left[-\beta {\displaystyle \sum_{l=1}^k{\upvarepsilon}_{i_l}\left(\mathbf{X};{\mathbf{z}}_{i_l}\right)}\right]{\mathrm{dV}}_{{\mathbf{y}}_I}}\right\} $$

is the RFE containing only a subset of component interactions (here, ${V}_{y_I}$ is the volume of the subspace spanned by variables ${y}_{i_1},{y}_{i_2},\dots, {y}_{i_k}$).

The factors of the first order, *f* ^(1)^, correspond to the PMFs of isolated units (e.g., isolated amino-acid residues) or those between isolated pairs of units (e.g., pairs of interacting side chains), while factors of order 2 and higher correspond to the multibody or correlation terms. All of the factors depend on temperature, and this dependence increases with increasing factor order because of the increasing order of the first term in the generalized-cumulant expansion of this factor [46, 50]. In our approach, as opposed to other coarse-grained force fields, this temperature dependence is explicitly accounted for [50].

The contributions of the correlation terms to the PMF and thus their importance depend on how many secondary variables are shared between the component energies, ε_*i*_, included in a particular factor. If no secondary variables are shared, the corresponding factor is equal to zero. For polypeptide chains, the variables that are strongly shared between the factors are the angles *λ* for rotation of the peptide groups about the C^α^⋯C^α^ virtual bonds.

The factor expansion is truncated [46] to achieve a good compromise between the complexity of the force field and its ability to reproduce the structure and dynamics of the system. We found that the fourth-order expansion is sufficient for the UNRES force field [58]. For the neoclassical force fields, e.g., MARTINI [35, 38, 59], all long-range interactions are approximated by factors of order 1 (i.e., by the potentials of mean force of isolated pairs of sites), while factors of order 2 occur only in the torsional potentials (these factors account for the coupling between the conformational states of the consecutive polymer units [46]). Approximate analytical formulae for factors can be obtained by taking the first nonzero generalized cumulant of its expansion into a generalized-cumulant series (which is very useful for correlation terms) [46] or by adapting the expressions from all-atom force fields (for the first-order factors and torsional potentials). These analytical expressions must be parameterized and the whole force field calibrated to reproduce the structure and physical properties of the system under study.

**Scheme 1:**
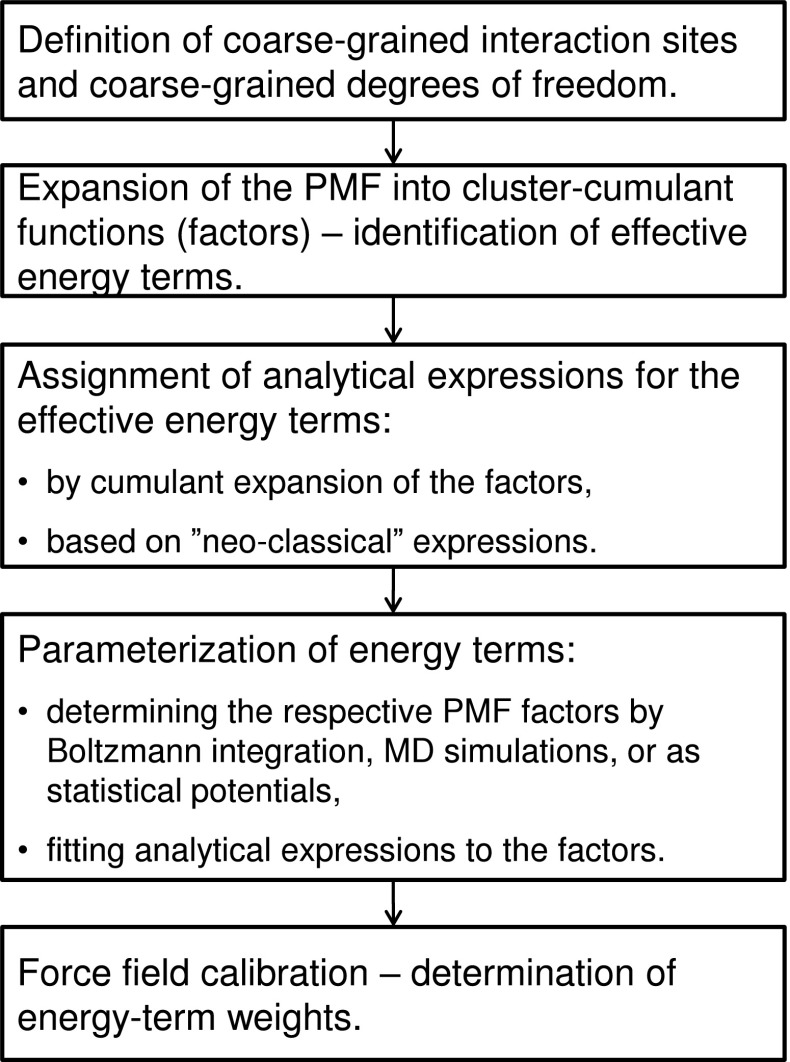
General scheme of the construction and parameterization of coarse-grained force fields based on Kubo cluster-cumulant expansion of the PMF

A general scheme of the construction of coarse-grained force fields based on the cluster-cumulant-expansion approach of the PMF is shown in Scheme 1.

### The UNRES model of polypeptide chains

In our UNRES model [46–54] (Fig. 1a), a polypeptide chain is represented by a sequence of α-carbon (C^α^) atoms linked by virtual bonds with attached united side chains (SC) and united peptide groups (p) located midway between the consecutive α-carbon (C^α^) atoms (Fig. 1a). Only the united peptide groups and united side chains act as interaction sites. The C^α^ atoms serve only to define the geometry of the backbone trace, and are not interaction sites in the UNRES model.

The energy of the virtual-bond polypeptide chain is expressed by

6 $$ U={w}_{\mathrm{S}\mathrm{CSC}}{\displaystyle \sum_j{\displaystyle \sum_{i< j}{U}_{\mathrm{S}{\mathrm{C}}_i\mathrm{S}{\mathrm{C}}_j}}}+{w}_{\mathrm{S}\mathrm{Cp}}{\displaystyle \sum_j{\displaystyle \sum_{i\ne j}{U}_{\mathrm{S}{\mathrm{C}}_i{\mathrm{p}}_j}}}+{f}_2(T){w}_{\mathrm{el}}{\displaystyle \sum_j{\displaystyle \sum_{i< j-1}{U}_{{\mathrm{p}}_i{\mathrm{p}}_j}}}+{f}_2(T){w}_{\mathrm{tor}}{\displaystyle \sum_i{U}_{\mathrm{tor}}\left({\gamma}_i\right)}\kern2em +{f}_3(T){w}_{\mathrm{tor}\mathrm{d}}{\displaystyle \sum_i{U}_{\mathrm{tor}\mathrm{d}}\left({\gamma}_i,{\gamma}_{i+1}\right)}+{w}_b{\displaystyle \sum_i{U}_{\mathrm{b}}\left({\theta}_i\right)}+{w}_{\mathrm{rot}}{\displaystyle \sum_i{U}_{\mathrm{rot}}\left({\alpha}_{\mathrm{S}{\mathrm{C}}_i},{\beta}_{\mathrm{S}{\mathrm{C}}_i}\right)}+\kern2em {\displaystyle \sum_{m=2}^{N_{\mathrm{corr}}}{f}_m(T){w}_{\mathrm{corr}}^{(m)}{U}_{\mathrm{corr}}^{(m)}}+{f}_3(T){w}_{\mathrm{turn}}^{(3)}{U}_{\mathrm{turn}}^{(3)}+{f}_4(T){w}_{\mathrm{turn}}^{(4)}{U}_{\mathrm{turn}}^{(4)}+{w}_{\mathrm{b}\mathrm{ond}}{\displaystyle \sum_i{U}_{\mathrm{b}\mathrm{ond}}\left({d}_i\right)}\kern2em +{w}_{\mathrm{S}\mathrm{S}}{\displaystyle \sum_{\mathrm{disulfide}\;\mathrm{bonds}}{U}_{\mathrm{S}{\mathrm{S}}_i}+{n}_{\mathrm{S}\mathrm{S}}{E}_{\mathrm{S}\mathrm{S}}}, $$

with

7 $$ {f}_n(T)= \ln \left[ \exp (1)+ \exp \left(-1\right)\right]/ \ln \left\{ \exp \left[{\left( T/{T}_{\circ}\right)}^{n-1}\right]+ \exp \left[-{\left( T/{T}_{\circ}\right)}^{n-1}\right]\right\} $$

The terms ${U}_{\mathrm{S}{\mathrm{C}}_i\mathrm{S}{\mathrm{C}}_j}$ correspond to the mean free energy of solvent-mediated interactions between the side chains. The terms ${U}_{\mathrm{S}{\mathrm{C}}_i{\mathrm{p}}_j}$ correspond to the excluded-volume potential of the side chain–peptide group interactions. The terms ${U}_{{\mathrm{p}}_i{\mathrm{p}}_j}$ represent the energy of mean-field electrostatic interactions between backbone peptide groups. The terms *U*_tor_ and *U*_tord_ are the torsional and double-torsional potentials, respectively, for rotation about a given virtual bond or two consecutive virtual bonds. The terms *U*_b_ and *U*_rot_ are the virtual-bond-angle-bending and side-chain-rotamer potentials, respectively, and the term *U*_bond_ accounts for backbone and side-chain virtual-bond stretching [51, 60]. We recently [61] extended the backbone-virtual-bond stretching term to account for the *trans*–*cis* transition of peptide groups. The terms *U*_corr_^(*m*)^ and *U*_turn_^(*m*)^ correspond to the correlations (of order *m*) between peptide-group electrostatic and backbone-local interactions [46, 49]. The terms *U*_turn_^(*m*)^ (the “turn” terms) involve consecutive segments of the chain. The correlation terms are absolutely essential for reproducing regular secondary structures, such as α-helices and β-sheets [46, 62]. We found [58] that correlation terms of order 3 and 4 are sufficient for the force field to reproduce regular protein structures. The terms ${U}_{\mathrm{S}{\mathrm{S}}_i}$ are the energies of distortion of disulfide bonds from their equilibrium configuration, *E*_SS_ is the energy of formation of an “unstrained” disulfide bond in the chain (relative to the presence of two free cysteine residues), and *n*_SS_ is the number of disulfide bonds. The *w* terms are the weights of the respective energy terms. The multipliers *f*_*n*_(*T*) account for the temperature dependence of the dominant terms corresponding to the generalized-cumulant expansion of the PMF factors (Eq. 4); for a factor with a lowest nonzero cumulant of order *m*, the multiplier varies as 1/*T* ^*m* − 1^ with temperature [50]. For detailed expressions of the respective energy terms, the reader is referred to our earlier work [46–53].

All terms except ${U}_{\mathrm{S}{\mathrm{C}}_i\mathrm{S}{\mathrm{C}}_j}$ were determined by numerically computing the PMF surfaces of systems representing the corresponding PMF factors from the energy surfaces calculated by ab initio quantum mechanics (for *U*_tor_, *U*_tord_, *U*_pp_, *U*_corr_ [51], *U*_SS_ [63]) or semiempirical AM1 (for *U*_b_ [60] and *U*_rot_, and *U*_vib_ [60]) energy surfaces and fitting the respective analytical expressions to them. We initially [64] determined the side chain–side chain interaction potentials as knowledge-based potentials from the Protein Data Bank (PDB); however, they were recently [53, 65–68] re-determined from the PMFs of models of pairs of amino-acid side chains in water from all-atom MD simulations in explicit water.

To determine the energy-term weights (the *w* terms in Eq. 6), we developed [50, 58, 69] a hierarchical optimization approach in which the objective is to fit the weights so as to reproduce the order of structure formation and the thermodynamics of thermal folding/unfolding of the proteins selected for calibration.

### The NARES-2P model of nucleic acids and the model of protein–nucleic acid interactions

In the NARES-2P model, depicted in Fig. 1b, a polynucleotide chain is represented by a sequence of virtual sugar (S) atoms that are located at the geometric centers of the sugar rings and linked by virtual bonds with attached united sugar bases (B) and united phosphate groups (P). These united sugar bases and the united phosphate groups serve as interaction sites. The energy of the virtual-bond chain is expressed by

8 $$ U={w}_{\mathrm{B}\mathrm{B}}^{\mathrm{GB}}{\displaystyle \sum_i{\displaystyle \sum_{j< i}{U}_{{\mathrm{B}}_i{\mathrm{B}}_j}^{\mathrm{GB}}+{w}_{\mathrm{B}\mathrm{B}}^{\mathrm{el}}}}{\displaystyle \sum_i{\displaystyle \sum_{j< i}{U}_{{\mathrm{B}}_i{\mathrm{B}}_j}^{\mathrm{el}}+{w}_{\mathrm{P}\mathrm{P}}}{\displaystyle \sum_i{\displaystyle \sum_{j< i}{U}_{{\mathrm{P}}_i{\mathrm{P}}_j}+{w}_{\mathrm{P}\mathrm{B}}}{\displaystyle \sum_i{\displaystyle \sum_{j\ne i}{U}_{{\mathrm{P}}_i{\mathrm{B}}_j}+}}}}{w}_{\mathrm{b}\mathrm{ond}}{\displaystyle \sum_i{U}_{\mathrm{b}\mathrm{ond}}\left({d}_i\right)+{w}_{\mathrm{b}} i}{\displaystyle \sum_i{U}_{\mathrm{b}}\left({\theta}_i\right)+{w}_{\mathrm{tor}}{f}_2(T)}{\displaystyle \sum_i{U}_{\mathrm{tor}}\left({\gamma}_i\right)+{w}_{\mathrm{rot}}}{\displaystyle \sum_i{U}_{\mathrm{rot}}\left({\alpha}_i,{\beta}_i\right)}, $$

where ${U}_{{\mathrm{B}}_i{\mathrm{B}}_j}^{\mathrm{GB}}$ denotes the nonbonded interactions between the coarse-grained sugar-base sites, which is described by the Gay–Berne anisotropic potential [70], ${U}_{{\mathrm{B}}_i{\mathrm{B}}_j}^{\mathrm{el}}$ denotes the mean-field interactions between nucleic-acid-base dipoles (similar to that between peptide groups in UNRES [47]), ${U}_{{\mathrm{P}}_i{\mathrm{P}}_j}$ denotes the mean-field potential of phosphate group interactions, which consists of a Debye–Hückel term to account for solvent- and counterion-mediated charge–charge interactions [71], and the Lennard–Jones term ${U}_{{\mathrm{P}}_i{\mathrm{B}}_j}$ denotes the excluded-volume potential of the interactions of phosphate groups with sugar-base centers, the role of which is to prevent the collapse of these sites on each other, while *U*_bond_, *U*_b_, *U*_tor_, and *U*_rot_ account for virtual-bond stretching, virtual-bond-angle bending, the energetics of rotation about the S⋯S virtual bonds, and the energetics of the local geometric states of sugar-base sites. No correlation terms, except for the torsional potentials, are present in the current version.

The terms ${U}_{{\mathrm{B}}_i{\mathrm{B}}_j}^{\mathrm{GB}}$ and ${U}_{{\mathrm{B}}_i{\mathrm{B}}_j}^{\mathrm{elec}}$ were determined by numerical integration of the respective all-atom energy surfaces calculated with the AMBER force field, as done in our early work on the UNRES potential [47], and were fitted to the respective analytical expression. The dominant term was found to account for the mean-field interactions of the dipole-moment component parallel to the axis of one base with the dipole-moment component of the second base, which is perpendicular to its axis. This term has a minimum when the two base axes are perpendicular to each other, which is close to the geometry of the Watson–Crick base pairs. The local terms were determined as knowledge-based potentials from nucleic acid structures. The multipliers *f*_*n*_(*T*) are defined by Eq. 7. In the current version, ${w}_{{\mathrm{B}}_i{\mathrm{B}}_j}^{\mathrm{GB}}=0.5$ and the other weights were set to 2 to achieve the physiological melting temperature.

### Coarse-grained model of polysaccharides (SUGRES-1P)

The sugar model developed in this project (depicted schematically in Fig. 1c) is a single-center model in which the anchor points are the glycosidic oxygen atoms (usually 1 and 4), with the sugar interaction site positioned between them. The ignored degrees of freedom are the rotation angles of the sugar rings about the O⋯O virtual bonds, usually the O(1)⋯O(4) virtual bonds, as seen in the structures of, e.g., cellulose and starch. Thus, the resulting force field has a component arising from mean-field backbone–dipole interactions that are averaged in the context of local interactions, just as for the UNRES model, and with the same functional forms [46]. Off-1,4 connections (the 1,2, 1,3, 1,6, etc. connections), including chain branching, fix the plane of the sugar ring involved; this rotational restriction is analogous to that imposed by the pyrrolidine ring in proline.

The current version of the SUGRES-1P model was developed for polysaccharides with 1,4-glycosidic bonds and parameterized for α- and β-D-glucose.

9 $$ U={w}_{\mathrm{S}\mathrm{S}}^{\mathrm{GB}}{\displaystyle \sum_i{\displaystyle \sum_{j< i}{U}_{{\mathrm{S}}_i{\mathrm{S}}_j}^{\mathrm{GB}}+{w}_{\mathrm{S}\mathrm{S}}^{\mathrm{el}}{f}_2(T)}{\displaystyle \sum_i{\displaystyle \sum_{j< i}{U}_{{\mathrm{S}}_i{\mathrm{S}}_j}^{\mathrm{el}}}}}\kern2em +{w}_{\mathrm{corr}}^{(3)}{f}_3(T){\displaystyle \sum_i{\displaystyle \sum_{j< i}{U}_{\mathrm{corr};{\mathrm{S}}_i{\mathrm{S}}_j}^{(3)}+{w}_{\mathrm{turn}}^{(3)}{f}_3(T)}{\displaystyle \sum_i{\displaystyle \sum_i{U}_{\mathrm{turn};{\mathrm{S}}_i{\mathrm{S}}_{i+2}}^{(3)}+{w}^{(4)}{U}_{\mathrm{corr}}^{(4)}}}}+{w}_{\mathrm{b}\mathrm{ond}}{\displaystyle \sum_i{U}_{\mathrm{b}\mathrm{ond}}\left({d}_i\right)+{w}_{\mathrm{b}}}{\displaystyle \sum_i{U}_{\mathrm{b}}\left({\theta}_i\right)+{w}_{\mathrm{tor}}{f}_2(T)}{\displaystyle \sum_i{U}_{\mathrm{tor}}\left({\gamma}_i\right)}, $$

where the terms ${U}_{{\mathrm{S}}_i{\mathrm{S}}_j}^{\mathrm{GB}}$ represent the mean-field van der Waals and solvent-mediated interactions between sugar rings, which are represented by the anisotropic Gay–Berne potential [70], ${U}_{{\mathrm{S}}_i{\mathrm{S}}_j}^{\mathrm{el}}$ represent the mean-field interactions of the sugar-ring dipoles outside of the context of local interactions (the same functional form as used for backbone peptide groups is applied [47]), ${U}_{\mathrm{corr};{\mathrm{S}}_i{\mathrm{S}}_j}^{(3)}$ and ${U}_{\mathrm{turn};{\mathrm{S}}_i{\mathrm{S}}_{i+2}}^{(3)}$ denote the correlation contributions that account for the restricted rotation of sugar-ring dipoles (again, the same functional forms are used as those employed for polypeptide chains [46, 49]), *U*_corr_^(4)^ is the sum of fourth-order correlation terms adapted from UNRES [48], *U*_bond_, *U*_b_, and *U*_tor_ denote the virtual-bond-deformation, virtual-bond-angle-deformation, and virtual-bond-torsional energies, respectively, and the *w* terms are the weights of the energy terms.

In the current preliminary version of SUGRES-1P, the parameters of ${U}_{{\mathrm{S}}_i{\mathrm{S}}_j}^{\mathrm{GB}}$ and ${U}_{{\mathrm{S}}_i{\mathrm{S}}_j}^{\mathrm{el}}$ were determined by calculating the potential energy surfaces as functions of the distance between sugar-ring centers and their orientation using the AM1 method of molecular quantum mechanics. These potential energy surfaces were then used to compute the potentials of mean force, by averaging out the rotation about the O(4)⋯O(4) virtual-bond axes, as done in our earlier work on the derivation of the ${U}_{{\mathrm{p}}_i{\mathrm{p}}_j}$ potentials for polypeptide chains [47, 49]. Therefore, the present version of SUGRES-1P can treat fibrillar polysaccharides which may contain only solitary water molecules inside. To include water, long-range interaction potentials were determined from molecular dynamics simulations using the same procedure as employed to determine the side chain–side chain interaction potentials [53]. The local-interaction parameters were determined from the PMFs of trisugars composed of all possible combinations of α- and β-D-glucose; these energy surfaces were subsequently used to compute the virtual-bond-torsional and virtual-bond-valence potentials using the procedures developed for the parameterization of UNRES [49, 72]. Two-dimensional Fourier series were also fitted to the energy surfaces of trisugars to derive the initial approximations of the parameters of the ${U}_{\mathrm{corr};{\mathrm{S}}_i{\mathrm{S}}_j}^{(3)}$ and ${U}_{\mathrm{turn};{\mathrm{S}}_i{\mathrm{S}}_{i+2}}^{(3)}$ correlation terms, as done in our earlier work on UNRES [46]. No further refinement of these parameters has been carried out so far.

### Implementation of the components of UCGM

The UNRES model was initially used with the conformational space annealing (CSA) method of global optimization [73] to predict protein structures as global minima of the potential energy function. To extend its applications, we later implemented Langevin dynamics with UNRES [39, 74]. The equations of motion for the UNRES chain are Langevin dynamics equations because the solvent is implicit in UNRES. Consequently, it contributes to conservative forces (through the RFE) and gives rise to nonconservative forces which originate in the energy exchange of the polypeptide chain with the solvent (the stochastic and friction forces). Because the geometry of an UNRES chain is not uniquely defined by the Cartesian coordinates of the interacting sites, we chose the virtual-bond vectors (C^α^⋯C^α^ and C^α^⋯SC) as generalized coordinates **q** and implemented the Lagrange approach to derive the equations of motion [39, 75, 76].

To enable larger MD steps (up to 20 fs, compared to the 1–2 fs time-step size applied in typical MD programs such as AMBER [77]), we have also designed the adaptive multiple time-step integration algorithm (A-MTS) [74].

To sample the conformational space more efficiently than achievable by canonical MD, we extended [78, 79] the UNRES/MD approach to the multiplexed replica-exchange molecular dynamics method (MREMD) [80].

The reader is referred to our earlier works on MD [39, 74–76] and REMD/MREMD [78, 79] implementations of UNRES.

We recently [81] parallelized the energy and force evaluations, which enabled us to run calculations of >500-residue proteins in a few days with massively parallel systems. To compute the averages from the results of simulations carried out at different temperatures, we adapted [50] the histogram-reweighting technique known as the weighted histogram analysis method (WHAM) [82]. With these extensions, we were able to calculate thermodynamic and ensemble-averaged structural characteristics of protein folding [50] and develop a physics-based protocol for protein-structure prediction in which the candidate predictions are conformations averaged over subensembles of structures with the highest probability below the folding-transition temperature [50].

The NARES-2P and SUGRES-1P models were built into the UNRES/MD platform and thus enabled us to carry out canonical [39] and replica-exchange [79] simulations of nucleic acids and polysaccharides, respectively.

The UNRES package, with full documentation, is available to the academic community at http://www.unres.pl. It will be extended to incorporate NARES-2P and SUGRES-1P as soon as these components are fully developed and parameterized. The current versions of NARES-2P and SUGRES-1P can be obtained from the authors on request.

## Results

In this section, we briefly summarize the results obtained with UNRES and the results of initial test calculations obtained with NARES-2P and SUGRES-1P.

As mentioned in the “Methods” section, the initial application of UNRES was to make energy-based predictions of protein structures, in which the native structure was sought as the global minimum in the effective energy surface [73]. Using this approach, we scored the best prediction of target T0063 (HDEA) [83] in the Third Community Wide Experiment on the Critical Assessment of Techniques for Protein Structure Prediction (CASP3) (see http://www.predictioncenter.org for more information about the CASP exercises). After implementing MD [39, 74, 76] and its extensions [79] in UNRES, we used a much better justified ensemble-based approach to prediction in which candidate predictions are sought as ensembles of geometrically similar structures [50]. Using this approach, we predicted correctly, as one of the only two groups, domain packing for the CASP10 target T0063 [84]; our prediction was featured by the CASP10 assessors. Based on sequence similarity, T0063 was a template-based modeling target but template-based methods failed to predict correct domain packing. Our predicted structure of this target is compared with the experimental structure in Fig. 3.

**Fig. 4:**
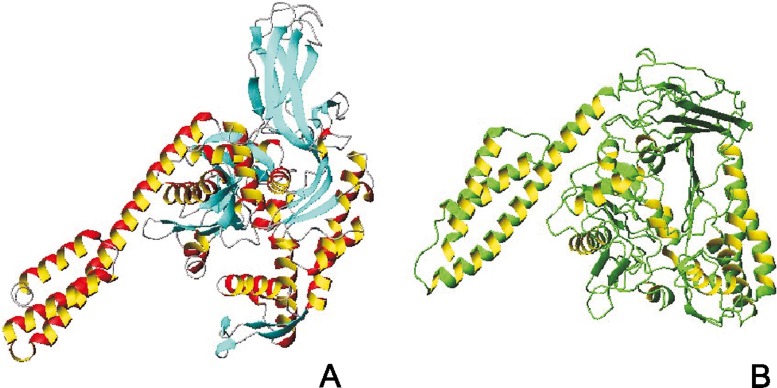
**a** The experimental structure [94] of the open conformation of DnaK (a bacterial chaperone, PDB: 1BQ9) and **b** the structure simulated [93] with UNRES/MD, starting from the closed (substrate-binding) conformation of the chaperone, before the experimental structure was determined

With the MD implementation of UNRES, we carried out extensive studies of protein folding, including a simulation of the kinetics of the folding of the B-domain of staphylococcal protein A [85], a description of the folding pathway of protein A obtained through network analysis [86], and free-energy landscapes of protein A and the FBP28 WW domain and its variants [87–89]. We also applied the UNRES/MD approach to determine the mechanisms of biophysical processes, including amyloid formation and growth [90, 91] as well as signaling [92], and to investigate the Hsp70 chaperone cycle [93]. In particular, with UNRES, we simulated the transition between the substrate-binding (closed) and ATP-bound (open) conformations of DnaK, a bacterial Hsp70 chaperone [93]. The open structure calculated by UNRES turned out to be very similar to the ATP-bound structure of DnaK solved one year later [94]. Our calculated structure is compared with the experimental structure in Fig. 4.

For small proteins, UNRES/MREMD calculations require only several hours to achieve the convergence of ensemble averages; for example, for the 46-residue fragment of the B-domain of staphylococcal protein A (a three-α-helix-bundle structure; this is one of the benchmark systems for UNRES calculations), 20 million MD steps per trajectory are run in about 7 h with Intel Pentium processors, with one core handling one trajectory. For larger systems (200–300 residue proteins), the same number of steps require about 24 CPU hours, with 4–16 cores handling one trajectory. A detailed study of the speed of UNRES and its parallel efficiency can be found in our earlier work [81].

Among the other physics-based force fields, the optimized potential for protein structure prediction (OPEP) from the Derreumaux group, which uses a detailed all-atom representation of the protein backbone and united side chains, was applied in ab initio folding. The latest version of the force field succeeded in folding the tryptophan zipper and the FBP28 WW domain (a three-stranded antiparallel β-sheet protein); the root-mean-square deviation (RMSD) of the most populated cluster was 3.8 Å [15, 16, 95]. This resolution, when scaled by protein size, is comparable to the resolution of the UNRES force field, although UNRES has been tested with larger sets of small proteins [50, 69, 96] and was also tested with larger proteins in the CASP experiments [52, 56, 83, 97, 98]. Just like UNRES [90, 91], OPEP was successfully used to simulate the aggregation of amyloidogenic peptides [3, 10, 11, 15].

**Fig. 5:**
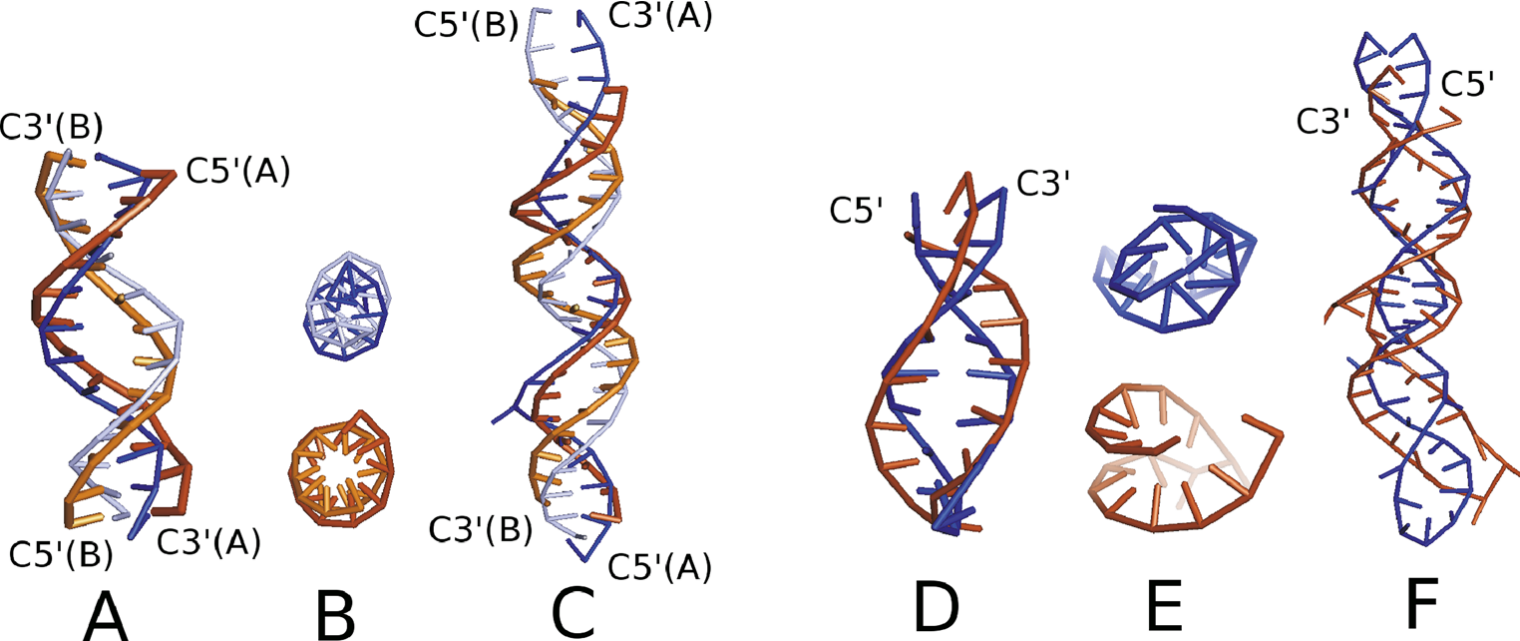
**a**–**f** Calculated ensemble-averaged structures at *T* = 300 K obtained in MREMD simulations (*thin blue sticks*) of the two small DNA molecules 9BNA (**a** and **b**) and 2JYK (**c**) and of the two small RNA molecules 2KPC (**d** and **e**) and 2KX8 (**f**), as compared to the respective experimental structures (*thick brown sticks*). For the *side views* (presented for all molecules), the calculated structures are superposed on the experimental structures, while the experimental structures are shown below the calculated structures for the *top views* (presented for 9BNA and 2JYK). The root-mean-square deviations (RMSDs) over the S centers averaged over all native-like clusters are 4.5 Å, 8.1 Å, and 5.7 Å for 9BNA, 2JYK, and 2KPC, respectively, and 9.8 Å for 2KX8 with respect to each experimental structure. The lowest RMSD values obtained in the respective MREMD runs are 2.9 Å, 5.6 Å, 1.6 Å, and 6.9 Å for 9BNA, 2JYK, 2KPC, and 2KX8, respectively. Reproduced with permission from Figure 2 of [56]

NARES-2P has not yet been applied to solve practical problems; however, we carried out extensive tests of this approach [56]. To assess the predictive power of NARES-2P, unrestricted multiplexed replica exchange simulations, started from extended unpaired chains, were carried out with two small DNA molecules (9BNA, 2 ×12 nucleotides; and 2JYK, 2 × 21 nucleotides) and two RNA molecules (2KPC, 17 nucleotides; 2KX8, 44 nucleotides) molecules. The conformational ensembles below the melting temperature consisted almost exclusively of native right-handed double-helical structures. Example results are shown in Fig. 5.

The coarse-grained DNA model from the Ouldridge group, which uses Morse-like potentials to reproduce base pairing and stacking with base-pair-type specific parameters [25, 30], and the HiRe-RNA model from the Derreumaux group [3, 28, 32], which uses Gaussian-type multibody terms to account for base pairing, can also fold nucleic acid molecules. However, both of these contain more interaction sites per nucleotide unit, and the functional forms of the potentials have been constructed to reproduce base pairing and stacking, while these features arise in NARES-2P from the mean-field electrostatic nature of the dominant base–base interaction terms. In addition, a number of statistical potentials [23, 24] reproduce the experimental RNA structures in ab initio folding simulations.

It is very interesting that removing or reducing the ${U}_{{\mathrm{B}}_i{\mathrm{B}}_j}^{\mathrm{elec}}$ component destroyed the folding capability of the NARES-2P force field, while removing local interactions (even the virtual-bond-angle terms) did not impair the ability of the force field to form double helices. Only right- and left-handed double helices appeared in comparable amounts due to the absence of the torsional potential that defines chain chirality [56]. These results suggest that the mean-field dipole–dipole interactions help to form structure. Unlike for proteins, the related correlation interactions do not appear to be required to reproduce double-helical structure.

**Fig. 6:**
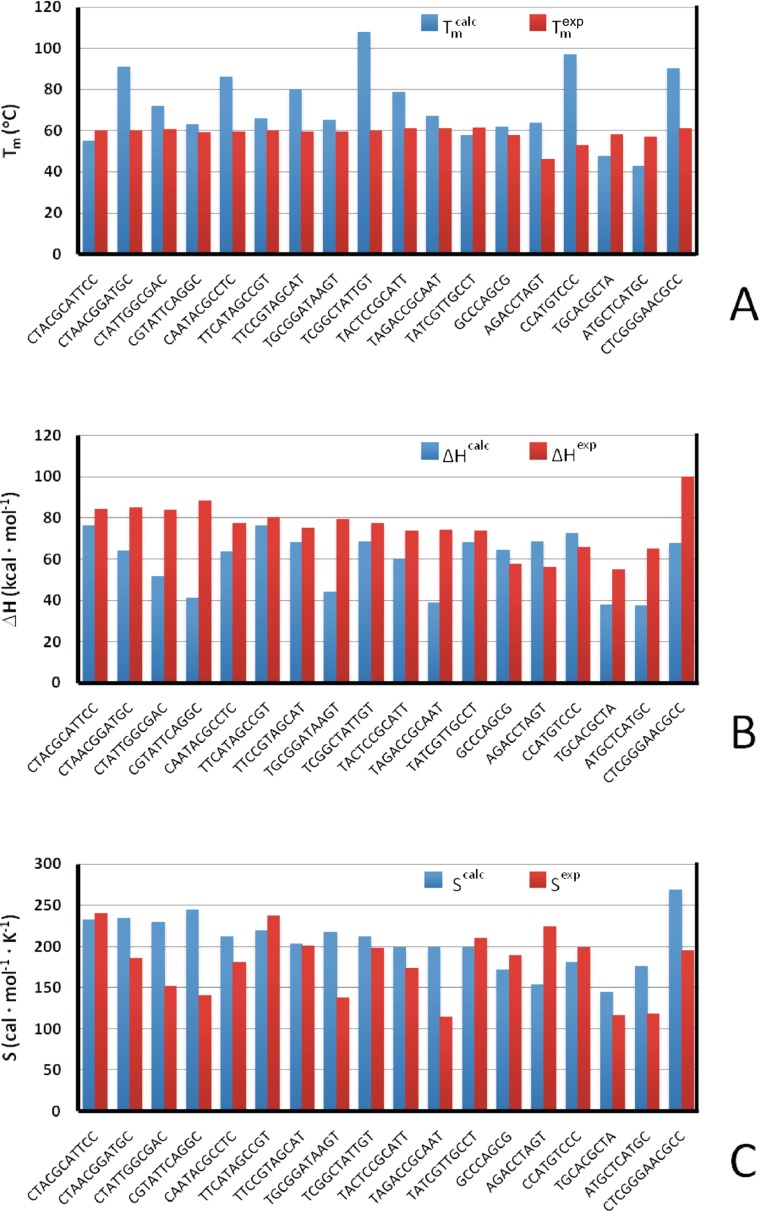
**a**–**c** Comparison of the experimental (*red bars*) and calculated (*blue bars*) temperatures (**a**), enthalpies (**b**), and entropies (**c**) of melting of model small DNA molecules. Data from [56]

We have also tested the ability of NARES-2P to reproduce the thermodynamic parameters associated with DNA melting. To accomplish this, we ran [56] MREMD simulations of a number of small DNA molecules for which the thermodynamics of melting were studied by calorimetry [99, 100]. As shown in Fig. 6, the agreement between the calculated and experimental melting temperatures, enthalpies, and entropies of melting is reasonable.

Because the NARES-2P energy function is less computationally expensive than the UNRES energy function (it does not have correlation terms), NARES-2P requires less time for a given number of MD steps. For example, for the 2KX8 RNA molecule (44 nucleotides), 20,000,000 MD steps take only 3 h. On the other hand, because the bases are usually mispaired in the initial folding stages and have to rearrange, it takes three- to fourfold more MD steps to obtain converged conformational averages as compared to the UNRES simulations for proteins.

**Fig. 7:**
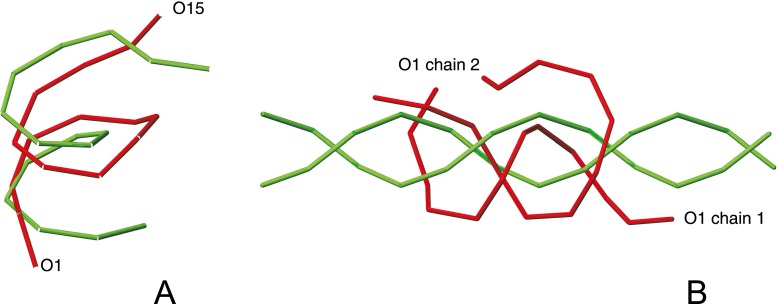
**a** Superposition of the experimental (*green*) and calculated (using the SUGRES-1P model; *red*) O4 traces of part of cyclic amylose. The RMSD over the O4 atoms is 5.5 Å. **b** Superposition of the experimental (*green*) and calculated (using the SUGRES-1P model; *red*) O4 traces of amylose A (structure from the PolySac3DB database [101]; http://polysac3db.cermav.cnrs.fr/polysacdb/amylose-a/AmyA_double.pdb). Calculations were carried out on the dimer composed of two 12-residue monomers. The RMSD over the O4 atoms is 7 Å. It can be seen that, despite considerable distortion of the calculated structure at the ends, the right-handed-twisted double-helical structure and parallel packing of the chains is preserved

The SUGRES-1P force field is at the initial development stage. Nevertheless, the limited tests carried out so far are encouraging. In Fig. 7, the average structure of the most populated cluster of conformations of a helical section of cyclic amylose, and that of a dimer of two 12-residue α-D-glucose chains (a unit of amylose), obtained in unrestricted MREMD simulations using the SUGRES-1P force field, are compared with the respective experimental data. As shown, the force field is able to reproduce the double-helical fold of both systems.

## Conclusions and outlook

The examples illustrated in the “Results” section have shown that it is possible to construct a unified coarse-grained model with a very small number of interaction sites per unit that describes the structure and energetics of proteins, nucleic acids, and polysaccharides surprisingly well. The success of the UCGM most probably results from two principles of its design: (i) the origin of the effective energy function in the potential of mean force, which is then split into factors, enabling us to extract pure components pertaining to a given part of the system under consideration without the danger of counting the same contributions multiple times, and (ii) focusing on electrostatic and local interactions between polar units, the interactions of which seem to determine biopolymer architecture. Moreover, all three components of the model are based on the same geometric design: placing backbone sites between two anchor points and attaching branches (side chains, nucleic acid bases) to the same anchor points. Therefore, merging all of the components of UCGM into one system is a relatively simple task. In particular, it is feasible to interface the oligosaccharide part to a protein to form the respective glycan. At present, we are also extending the model to protein–nucleic acid interactions. We have already developed the potentials of interactions between protein side chains and nucleic acid bases (Yin Y, Sieradzan AK, Liwo A, He Y, Scheraga HA, manuscript in preparation). Using these extensions, the model will become a tool with which it will be possible to study the energetics and dynamics of biochemical processes using a small fraction of the computational effort required by all-atom simulations, while still being able to keep track of the physics of the respective phenomena.

The transferability and universality resulting from maintaining close connections of the effective UNRES, NARES-2P, and SUGRES-1P energy functions with the physics of interactions in these types of macromolecules is the greatest advantage of the unified coarse-grained model.

On the other hand, the resolution of the components of the model, even the most advanced UNRES model for proteins, is only moderate (about 5 Å for an approx. 50-residue protein). Fortunately, this feature does not seem to be inherent in the coarse-grained approach because some of our test calculation resulted in average RMSDs of about 2 Å for a 67-residue protein [96]. The force fields constituting UCGM are probably still missing details of local interactions. Work on improving the representation of local interactions is in progress in our laboratory. Very recently [54], we introduced torsional potentials involving the virtual C^α^⋯SC bonds. This modification improved the resolution of the force field by about 0.5 Å on average [54].
